# Spontaneously Healed Pathologic Fracture over a Critical-Size Calcaneal Cyst

**DOI:** 10.1155/2011/861094

**Published:** 2011-08-04

**Authors:** Nikolaos G. Lasanianos, Ioannis Spanos, Aggeliki Papaioannou, Elisavet Paneri

**Affiliations:** 1st Department of Trauma & Orthopaedic surgery, Athens General Infirmary “Evaggelismos”, 10676 Athens, Greece

## Abstract

Simple bone cysts are nonsymptomatic lesions. They typically involve the medullary cavity, but they can also be found in nonlong bones such as the calcaneum. Their treatment remains controversial varying from observation and conservative healing to irritating injections or bone grafting. In the case of a pathologic fracture, surgical treatment seems most appropriate especially when the cyst is situated on a weight-bearing bone. We present herein the rare case of a spontaneously healed pathological fracture over a critical-size calcaneal cyst of a patient reluctant to undergo surgical treatment. An interpretation of the healing procedure as well as a review of the literature is presented.

## 1. Introduction

Unicameral bone cysts are lesions for which little is known concerning their aetiology and natural history. They are relatively common in the humerus, femur, and tibia, but quite uncommon in the calcaneum [1]. Calcaneal cysts are usually nonsymptomatic, and some of them are detected as incidental findings. Treatment of calcaneal cysts has been advocated based on the fear of pathologic fracture and collapse [2]. If conservative management has failed, other options include surgical management with curettage and bone grafting or with steroid injections [3–5]. The aforementioned methods may obliterate the cyst and anticipate a pathologic fracture. But what shall happen if a pathologic fracture already exists? In this case, surgical intervention with bone grafting [1, 2] or calcium phosphate bone cement [6], in order for the void to be filled and fragments of the fracture not to collapse, seems to be a one-way solution [7]. The surgical treatment of a pathologic fracture over a bone cyst is even more substantial when dealing with weight-bearing bones such as the calcaneum. Herein we report the rare case of a spontaneously healed pathologic fracture over a not healed calcaneal bone cyst which to the best of our knowledge has never been reported before. Conservative treatment was selected as a result of the reluctance of the patient to undergo the indicated surgical treatment. 

## 2. Case Report

**Figure 1:**
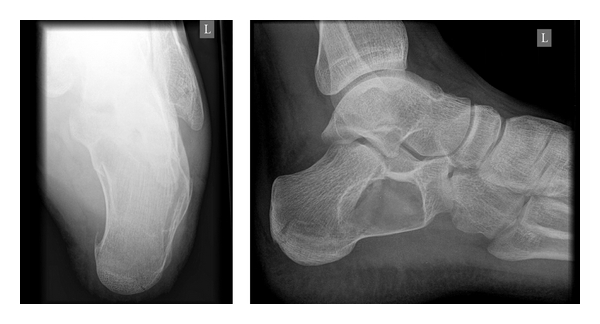
Axial and lateral plain X-rays showing a large radiolucent area in the anterior half of the calcaneal body resembling to a cyst. The lines of the pathologic fracture can also be observed.

**Figure 2:**
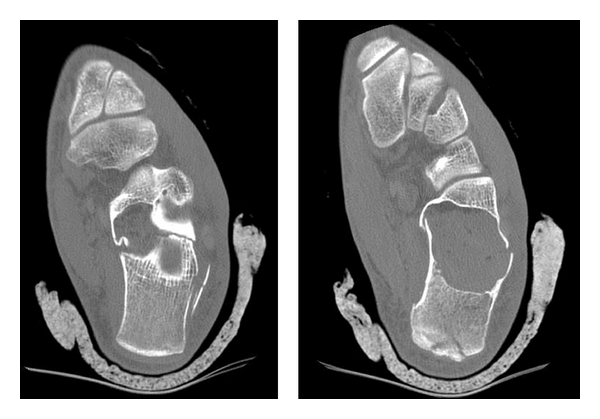
CT scan views on the transverse plane showing an undisplaced pathologic fracture over a simple calcaneal cyst.

**Figure 3:**
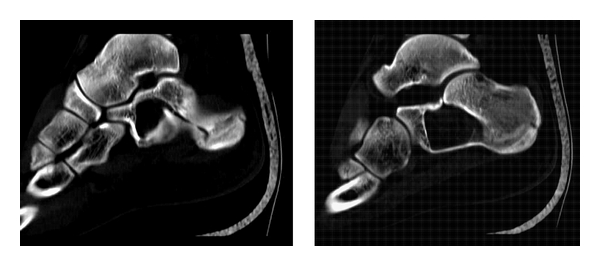
CT scan views on the sagittal plane showing an undisplaced pathologic fracture over a simple calcaneal cyst.

A 19-year-old man presented to the accident and emergency (A & E) department with a painful left heel after falling off from a height of approximately 1 m and landing on both feet. He was mobilizing with difficulty on toe-touch weight-bearing mobilization. His left hindfoot was swollen and extremely tender. Plain radiographs (lateral and axial views) of both feet were acquired. The right hindfoot proved to be free of any fracture or other pathology. The left hindfoot radiographs revealed a large radiolucent area in the anterior half of the calcaneal body resembling to a cyst (Figure 1). In the eyes of the on-call registrars, no fracture pathology was clearly evident at the time. Since the clinical view was quite aggravated, the calcaneum had a strange radiographic appearance, no definite diagnosis was set, and a computed tomography (CT scan) was requested. The CT scan revealed an undisplaced pathologic fracture over a 3.8 × 2.4 × 4.2 cm calcaneal simple cyst (Figures 2 and 3). The fracture of the calcaneum had a tongue type configuration extending to the junction of the anterior and posterior subtalar joint at the superior aspect of the calcaneal cyst. Both medial and lateral walls of the pinned out calcaneum were also fractured. The sustentacular process was involved by the cyst only at its base with a more mid-to-distal aspect completely normal. The cyst, which was void of bone, filled 100% of the intracalcaneal cross-section in the coronary plane and at least 50% in the sagittal plane.

**Figure 4:**
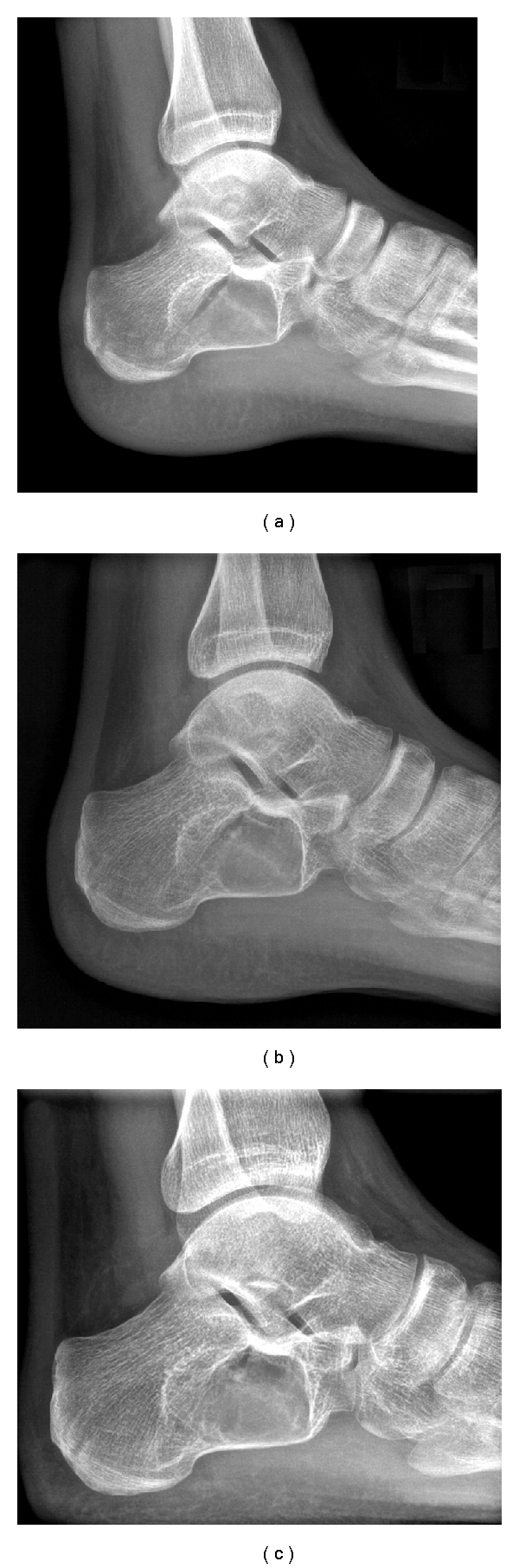
(a)Radiographic control 4 weeks after injury. (b)Radiographic control 8 weeks after injury. (c)Radiographic control 18 months after injury.

The patient was consulted to undergo surgical treatment for grafting and stabilization of his fracture. However, he did not consent to this and chose to be treated conservatively although he was warned about the possibility of improper healing and recurrent complications. After the patient's refusal to be admitted in the hospital, a short leg posterior slab was applied prior to discharge. He was advised to have ice therapy, foot elevation, and non-weight-bearing mobilization. The posterior slab was transformed to a below the knee jigsaw cast in 2 weeks, after the swelling had totally subsided. Radiographic control 4 weeks after injury (Figure 4(a)) showed signs of healing, and the patient was advised to start performing range of motion (ROM) exercises in order for ankle stiffness to be avoided. New X-rays, 8 weeks after injury (Figure 4(b)), revealed callus formation at the fracture site. This, in combination with the lack of pain or tenderness symptoms, drove us to the decision of allowing partial weight-bearing mobilization to be initiated. Full weight-bearing mobilization was allowed 12 weeks after injury. The patient was under monthly radiographic control (lateral and axial views) for the first 6 months remaining asymptomatic. He was discharged back to the care of his general practitioner and was advised to be clinically and radiographically checked on annual base. At his last followup 18 months (Figure 4(c)) after injury, he was painlessly ambulatory with no restriction to his ankle or foot ROM although there were no signs of mineralization in the cystic lesion. 

## 3. Discussion

Simple or unicameral bone cysts are benign fluid-containing skeletal lesions occurring most frequently in the first 2 decades of life [1, 7–11]. In children and adolescents, most cysts are in the proximal ends of the diaphysis of the humerus or femur [8–10]. Other locations such as calcaneal occur mostly in adults (with a prevalence of about 3%) [8, 10, 12, 13]. In this location, they are found at the base of the calcaneal neck at the inferior anterior margin of the posterior facet [1, 2]. The male to female ratio is approximately 2 : 1 [1, 12]. Most simple bone cysts of the calcaneum (60%) are asymptomatic and are found incidentally on radiographs for other conditions [1, 2]. They appear as well-circumscribed areas of oval lucency within the anterior portion of the calcaneum below the sulcus calcanei and the posterior articular surface for the talus (facies articularis talaris posterior). There may be a thin sclerotic rim around the lesion. There is minimal or no bony trabeculae transversing the cyst [1, 7, 14]. Heel pain is the presenting complaint in 38% of the cases [14]. 

A wide variety of treatment methods have been described for the healing of unicameral bone cysts, while there are no guidelines for their management [2, 13, 15]. R. W. Smith and C. F. Smith [1] treated conservatively patients with no symptoms or patients with activity-induced pain. Many authors recommend surgical treatment based on the fear of pathologic fracture [2, 7, 8, 16]. In Pogoda's study [7], operative treatment is recommended for symptomatic critical-size cysts (100% intracalcaneal cross-section in the coronary plane and at least 30% in the sagittal plane) because pathologic fracture is a significant consequence in this group. Treatment options vary from injections of steroids to multiple drill hole decompression, curettage and bone grafting, curettage combined with multiple drilling, and decompression with or without placement of Kirschner wires or screws [1–3, 7, 8]. Pathologic fracture of simple bone cysts is common and often leads to the classic plain film finding of the “fallen fragment” sign, which occurs in about 20% of cases [17]. Concerning the calcaneum, pathologic fractures are rare (2%) because of the usual positioning of the cysts in the non-weight-bearing region of the calcaneum [1, 2, 6–8, 11]. This may be the reason why a pathologic fracture with collapse has not been reported in cysts of the calcaneum although it is common in cysts found in other locations such as humerus and femur [2]. Nonetheless, collapse and deformity of traumatic calcaneal fractures in adults yields such poor functional results that surgeons universally hesitate recommending observation as the sole management of a significant-size cyst of the calcaneum no matter its positioning [2]. 

Based on the literature, conservative treatment was not a rational option in our case for two reasons: first, we had to deal with a critical-size cyst, based on Pogoda's description [7], and second, we had to manage a pathologic fracture over that cyst. However, we were obliged to the decision of not surgically interfering because of the patient's reluctance to be operated even though he was informed that his situation was expected to deteriorate and that delay of the operation would compromise the final result. Surprisingly, we were wrong, and the patient proved to be right. We never saw the “fallen fragment” sign as healing of his fracture spontaneously occurred and full weight bearing was accomplished 12 weeks after injury. It has been stated that simple bone cysts have a tendency to heal after fracturing, and in the long run, the fracturing itself is somewhat beneficial [18]. Davé et al. [19] reports the healing of a humeral bone cyst after a pathological fracture. In our case though, the fracture healed without healing of the cyst being accomplished simultaneously. 

It is notable that although some authors advocate operative treatment based on the risk of pathologic fracture [2, 4, 20–22], others disregard that the biomechanical strength of the calcaneum is compromised by simple bone cysts [23, 24]. We tend to agree with the latter opinion for the reason that although our patient suffered a fracture over his calcaneal cyst after a medial-height fall, his fracture was spontaneously healed, and moreover, he has been asymptomatic thereafter for the next 18 months. The paradoxical healing of the pathologic fracture without simultaneous cyst healing, in the case presented, may be an exception to the assumption that fracturing may be beneficial for the healing of simple bone cysts [18]. Moreover, one could think that conservative treatment in a cast might be optimal for pathologic calcaneal fractures. However, even after the experience of this case, we would still recommend surgical intervention to the next patient that would show up with a similar pathological fracture pattern. Further research is needed for definite conclusions to be drawn and for us to understand if our patient was safe or just lucky. A final point we believe should be made is that doctors of the A & E department shall always ask for a CT scan whenever a calcaneal cyst is observed on plain X-rays. Apart from revealing potentially occult pathologic fractures, as happened in this case, a CT scan shall also inform on the characteristics of the cyst indicating the most appropriate way of management.
